# A new species of the genus *Diplocentrus* Peters, 1861 (Scorpiones, Diplocentridae) from Oaxaca, Mexico

**DOI:** 10.3897/zookeys.412.7619

**Published:** 2014-05-29

**Authors:** Carlos Eduardo Santibáñez-López

**Affiliations:** 1 Colección Nacional de Arácnidos, Instituto de Biología, Circuito exterior s/n, Ciudad Universitaria, Copilco, Coyoacán A.P. 70-233, Distrito Federal, C.P. 04510, México

**Keywords:** Scorpions, diversity, *mexicanus* group

## Abstract

A new species of the genus *Diplocentrus* Peters, 1861 is described, based on several specimens collected in the Mexican state of Oaxaca. It is characterized by a high telotarsal spiniform setae count (4-5/5:5/6:6/6:6/6-7), and the pectinal tooth counts of 12–15, mode = 13 (male) or 11–13, mode = 12 (female). With the description of this species, the diversity of the genus is increased to 51 species in Mexico.

## Introduction

The genus *Diplocentrus* Peters, 1861 comprises nearly 60 species, 51 of them are distributed in Mexico, is the most diverse genus in the family Diplocentridae Karsch, 1880 (Santibáñez-López et al. 2013a). The Mexican species were divided in two groups by Hoffmann (1931), based on size and coloration. Francke (1977) redefined the groups in a key to identification of the *Diplocentrus* species occurring in the Mexican state of Oaxaca, based on cheliceral and pedipalp femur ratios, and renamed the *whitei* group to *mexicanus* group because it included type species (*Diplocentrus mexicanus* Peters, 1861). Nevertheless, Francke (1978) realized that the distinction of both groups was problematic because the diagnostic characters of the pedipalp femur were also used to separate other genera in the family. Recently, Santibáñez-López et al. (2013a) presented an operational diagnosis for the *keyserlingii* group; but did not assume that it was monophyletic, pending further investigation of *Diplocentrus* phylogeny. Fifteen species are reported for the Mexican state of Oaxaca, nine of them belong to the *keyserlingii* group, and six to the *mexicanus* group. In the present contribution, *Diplocentrus franckei*, sp. n. from the *mexicanus* group is described from Oaxaca, Mexico; it is compared to its most morphological similar species.

## Materials and methods

Scorpion higher classification follows Prendini and Wheeler (2005). Nomenclature and measurements follow Stahnke (1970), except for trichobotria (Vachon 1974), carination of the metasoma (Francke 1977), and pedipalps (Prendini 2000), and carapace surfaces (Prendini et al. 2003). Basitarsi spiniform macrosetae counts as explained in Santibáñez-López et al. (2013b)

Observations were made using a Nikon SMZ-800 stereomicroscope. Measurements, given in millimeters, were obtained with an ocular micrometer calibrated at 10X. Digital images were taken under visible and UV light with a Microptics ML-1000 digital imaging system, equipped with a Nikon DS80 camera, or a Nikon SMZ-800 with Nikon Coolpix S10 VR camera attachment. The focal planes of image stacks were fused with CombineZM (Hadley 2008) and composite images edited with Adobe Photoshop CS6. Distribution maps were generated in ArcView Ver. 3.2 (ESRI), using the locality coordinates, a base map from CONABIO (2011) digital database, and a digital elevation model from the CGIAR Consortium for Spatial Information (Jarvis et al. 2008). Geographical coordinates of collection localities were recorded in the field with a GARMIN eTREX H GPS device. Localities without geographical coordinates were retroactively georeferenced using the INEGI (2011) Archivo Histórico de Localidades dataset.

Abbreviations for depositories: AMNH – American Museum of Natural History, New York, USA; CNAN – Colección Nacional de Arácnidos, Instituto de Biología, Universidad Nacional Autónoma de México, DF, Mexico; CALA – Colección Institucional “Luis de Armas”, Instituto Tecnológico del Valle de Oaxaca, Oaxaca. CAIMSc. Colección de Artrópodos con importancia médica (CAIM) Laboratorio de Entomología, Instituto de Diagnóstico y Referencia Epidemiológicos (InDRE), Mexico.

## Taxonomic account

### Family Diplocentridae Karsch, 1880
Genus *Diplocentrus* Peters, 1861

#### Diplocentrus franckei sp. n.

http://zoobank.org/B219152E-7FEA-4EDC-985C-592C5C5B0422

http://species-id.net/wiki/Diplocentrus_franckei

**Figure 1.**
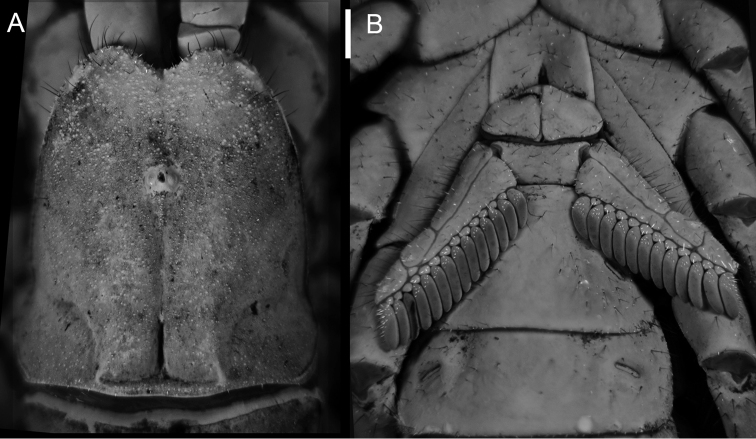
*Diplocentrus franckei* sp. n., **A** Carapace, dorsal aspect **B** sternum, genital operculum and pectines, ventral aspect.

Figures 1 [Fig F2] [Fig F3] [Fig F4] [Fig F5] –6

##### Type material.

Holotype: Male from MEXICO. OAXACA. Distrito de Villa Alta. Municipio de San Melchor Betaza (CNAN-T0668), km 101 road to Villa Alta 17°13.463'N, 96°09.124'W, 992 m., 21 June 2007, C. Santibáñez and A. Valdez. Paratypes one adult female, one subadult male, one juvenil male and one juvenil female (CNAN- T0669) (same data as holotype). One adult male, three adult females (CNAN-T0670) from San Melchor Betaza 17°15.061'N, 96°09.188'W, 1415 m., 1 June 2007, C. Santibáñez and H. Jara. One adult female, one adult male (AMNH), two adult males, six juvenil male, three adult females and six juvenile female (CNAN) from Municipio de San Andrés Zoolaga, 6 km south 17°15.4722'N, 96°14.3928'W, 1119 m., 21 June 2007, O. Francke, A. Ballesteros, H. Montaño, C. Santibáñez and A. Valdez.

##### Additional material.

MEXICO. OAXACA. Distrito de VILLA ALTA: Municipio de San Juan Tabaa, one adult female (CALA) [17°18.292'N, 96°12.390'W, 1280 m.], 10 June 2004, T. Martínez. One adult female (CALA) same data, 12 June 2004, T. Martínez. One adult male (CALA) from Municipio de San Francisco Yovego [17°33.4848'N, 96°13.551'W, 589 m.], 1 April 2005, T. Martínez. Two adult males (CNAN) from Municipio de San Melchor Betaza [17°15.061'N, 96°09.188'W, 1415 m.], June 2008, R. Mejía. One adult female (CNAN) from 9 km from San Andres Yaa on the road to San Juan Tabaa 17°20.1312'N, 96°11.2188'W, 787 m., 3. April 2007, C. Santibáñez and H. Jara. One adult female (CAIMsc-01136) from Municipio San Juan Yaeé, Santiago Yagallo [17°25.4166'N, 96°17.833'W, 1200 m.], 12 March 1997, P. Ruiz Figueroa.

##### Diagnosis.

**Figure 2. F2:**
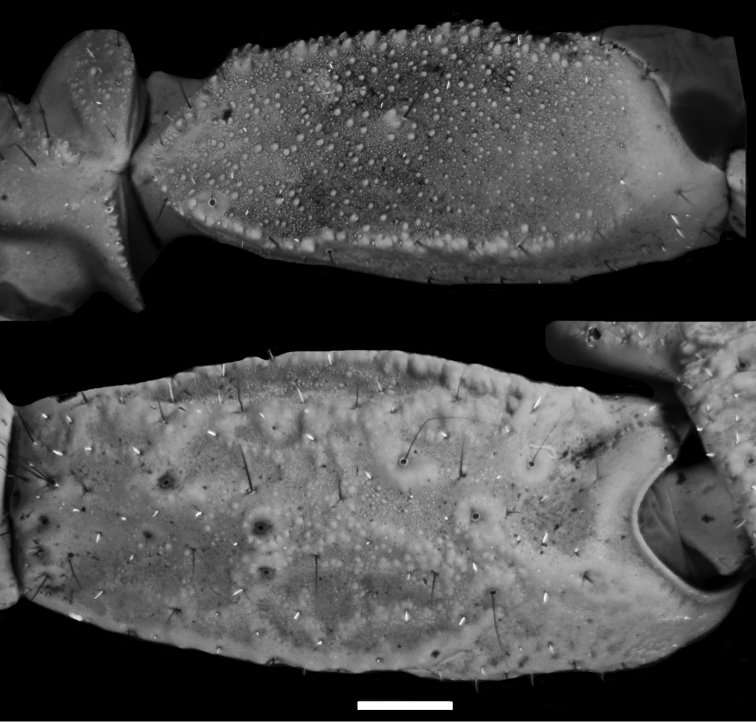
*Diplocentrus franckei* sp. n., **A** dextral pedipalp femur, dorsal aspect **B** dextral pedipalp patella, external aspect.

**Figure 3. F3:**
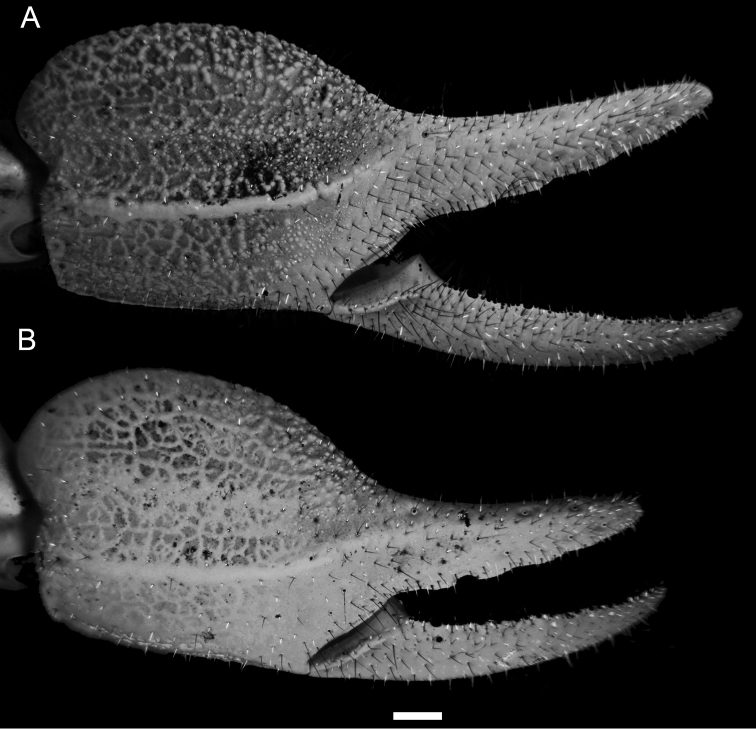
*Diplocentrus franckei* sp. n., dextral pedipalp chela, dorsoexternal aspect. **A** Holotype ♂ (CNAN) **B** Paratype ♀ (CNAN). Scale bar = 1 mm.

The following character combination is diagnostic for *Diplocentrus franckei*, sp. n. Total length (adult), 55 to 60 mm. Base coloration (adult) brown to dark brown. Carapace anteromedian notch moderately deep, U-shaped (Fig. 1A). Pedipalp femur, dorsal surface sparsely and finely granular (Fig. 2A). Pedipalp patella, dorsomedian carina moderately developed, crenulate to feebly granular (male); dorsoexternal strongly developed, crenulate to feebly granular (male); externomedian carina moderately developed, crenulate (male; Fig. 2B); ventromedian carina weakly developed to faint, granular (male, female). Pedipalp chela manus, dorsal surface markedly reticulate (male, Fig. 3A) or weakly reticulate (female, Fig. 3B); digital carina strongly developed, smooth to crenulate (male) or weakly to moderately developed, smooth (female); dorsal secondary carina weakly developed to faint, coarsely granular (male) or faint, smooth (female); dorsal external carina weakly developed to faint, smooth to crenulate (male), or faint, smooth (female). Legs I-IV telotarsi, counts of spiniform macrosetae in pro- and retroventral rows, 4-5/5:5/6:6/6:6/6-7 (variation in Table 1); basitarsi spiniform macrosetae pattern: leg I pst, rst, pm, rm; leg II pt, rt, pst, rst, pm, rm, Rm; legs III-IV pt, rt, vt, rst, vst, vm (variation in Table 2). Pectinal tooth count, 12-15, mode = 13 (male) or 11-13, mode = 12 (female) (variation in Table 3).

**Table 1.** Telotarsal spiniform macrosetal count (number of macrosetae in pro- and retroventral rows of telotarsi on legs I–IV) in *Diplocentrus franckei* sp. n., given as number of legs observed with corresponding proventral (p) and retroventral (r) setal count.

	Leg I				Leg II				Leg III					Leg IV				
	p		R		p		r		p		r			p			r	
	4	5	4	5	5	6	5	6	5	6	5	6	7	5	6	7	6	7
*Diplocentrus franckei* sp. n.	26	42	4	63	66	2	18	49	5	63	3	60	6	3	63	1	46	21

**Table 2.** Basitarsal spiniform macrosetal count (number of macrosetae of basitarsi on legs I-IV) in *Diplocentrus franckei* sp. n., given as numbers of legs observed with corresponding setae count.

Leg	n	pt	rt	vt	pst	rst	vst	pm	rm	vm	Rm
I	41	1	1		41	41		41	41		5
II	42	42	42		42	42		42	42		42
III	42	42	42	42		42	42			42	
IV	36	36	36	36		36	36			36	

**Table 3.** Pectinal tooth count (number of teeth per pecten) in *Diplocentrus franckei* sp. n., given as number of male and female pectines observed with corresponding tooth count.

		11	12	13	14	15	16
*Diplocentrus franckei* sp. n.	male	3	14	7	2		
	female	6	26	5	1		

*Diplocentrus franckei* sp. n. resembles *Diplocentrus mexicanus* Peters, 1861, *Diplocentrus melici* Armas et al., 2004 and *Diplocentrus jaca* Armas and Martín-Frías, 2000 in adult size and coloration, but can be distinguished as follows. The leg III telotarsi counts of spiniform macrosetae is higher in *Diplocentrus mexicanus* (7/8), while is lower in *Diplocentrus franckei*, sp. n. (6/6). The carapace and mesosoma surfaces are strongly granular (male) in *Diplocentrus mexicanus*, but weakly granulose to shagreened in *Diplocentrus franckei* (male). Metasomal and pedipalp carination is slightly granular to crenulate in *Diplocentrus mexicanus* (male), and crenulate to smooth in *Diplocentrus franckei* (male). The rt spiniform macroseta in leg I is present in *Diplocentrus mexicanus*, while it is absent in *Diplocentrus franckei*; also rsm in leg II is present in *Diplocentrus mexicanus* and absent in *Diplocentrus franckei*.

Pedipalp surfaces in *Diplocentrus melici* are punctuate, and minutely granular or smooth in *Diplocentrus franckei*. Carapace anteromargin notch U-shaped in *Diplocentrus franckei*, while in *Diplocentrus melici* is V-shaped. Pedipalp patella dorsoexternal and externomedian carinae are strongly developed in *Diplocentrus franckei* (male), but both are obsolete in *Diplocentrus melici* (male).

Adults of *Diplocentrus franckei* (55–60 mm) are smaller than adults of *Diplocentrus jaca* (75–90 mm). Metasomal segment V ventral carinae are strongly serrate in *Diplocentrus jaca*, while in *Diplocentrus franckei* are granular. Carapace anteromargin notch is strongly deep (reaching the second pair of lateral ocelli level) in *Diplocentrus jaca*, while it is moderately deep (reaching the first pair of lateral ocelli level) in *Diplocentrus franckei*. Pedipalp chela is slender in *Diplocentrus jaca* (male, chela length:ratio= 4.97), and rounded in *Diplocentrus franckei* (male, chela length:ratio= 2.32).

**Figure 4. F4:**
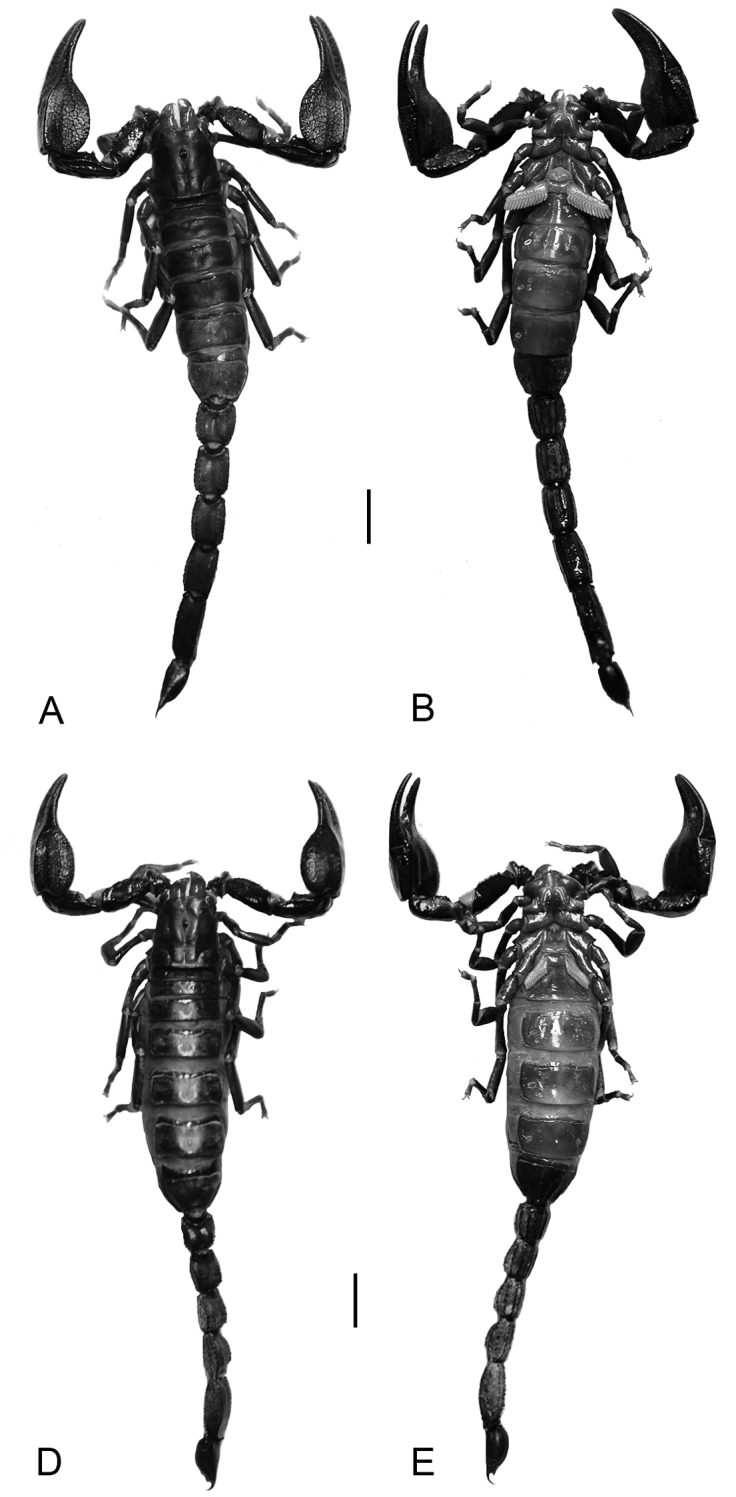
*Diplocentrus franckei* sp. n., habitus, dorsal (**A, C**) and ventral (**B, D**) aspect. **A, B.** Holotype ♂ (CNAN) **C, D** Paratype ♀ (CNAN). Scale bars = 5 mm.

**Description.** Based on holotype (male) and paratype (male) (Fig. 4A, B) with differences in paratype (female) (Figs 4C, D) noted. Measurements in Table 4.

**Table 4.** Measurements (mm) of male and female type specimens of *Diplocentrus franckei*, sp. n., in the CNAN.

	♂ Holotype	♀ Paratype
Total L	57.8	57.2
Carapace L	7.1	7.7
Carapace W	6.8	7.5
Mesosoma L	18.5	21.9
Pedipalp L	27.2	24.6
Femur L	6.1	5.5
W	2.2	2.4
D	2.0	2.0
Patella L	6.8	6.0
W	2.0	2.0
D	2.8	2.7
Chela L	14.3	13.1
W	3.0	3.7
D	6.2	6.1
Movable finger L	9.3	7.4
Fixed finger L	6.0	4.9
Chelicera L	3.2	3.2
W	1.4	1.6
Movable finger L	2.0	2.1
Fixed finger L	1.2	1.1
Metasoma L	27.0	22.5
Segment IV L	5.5	4.8
W	3.0	3.0
Segment V L	8.0	6.0
W	2.5	2.6
D	2.5	2.5
Telson L	5.2	5.1
Vesicle L	5.2	5.1
W	2.5	3.0
D	2.4	2.5

*Coloration*: Carapace brown (male) or darker brown (female), moderately infuscate throughout, uniformly around median ocelli, variegated elsewhere. Coxosternum pale yellow to light brown. Pedipalps brown to dark brown, carinae darker. Legs brown to reddish brown, uniformly and faintly infuscate. Mesosoma brown (male) to dark brown (female), tergites moderately (male) to weakly (female) infuscate; sternites pale brown to pale yellow. Metasoma reddish brown (male) to dark brown (female). Telson brown, uniformly infuscate.

*Carapace*: Anterior margin weakly setose; anteromedian notch weakly to moderately deep, U-shaped (Fig. 1A). Frontal lobes and interocular surface moderately granular; surfaces around median ocular tubercle shagreened; other surfaces minutely, sparsely and finely granular. Three pairs of subequal lateral ocelli.

*Pedipalps*: Orthobothriotaxic, Type C. Femur width greater than height (Fig. 2A); dorsal intercarinal surface flat, sparsely granular; external intercarinal surface smooth; ventral intercarinal surface flat, shagreened to minutely, finely granular; internal intercarinal surface coarsely and densely granular; dorsointernal carina strongly developed, comprising several large granules; dorsoexternal carina moderately to weakly developed, comprising few large granules; ventroexternal carina obsolete; ventrointernal carina moderately developed, comprising large granules. Patella, dorsal and external intercarinal surfaces slightly, minutely and finely granular-reticulate (Fig. 2B); ventral intercarinal surface flat, minutely granular between ventrointernal and ventromedian carinae, smooth to slightly reticulate between ventromedian and ventroexternal carinae (male) or smooth (female); internal intercarinal surface sparsely granular; proximal tubercle strongly developed, comprising two large granules; dorsointernal carina obsolete; dorsomedian carina moderately developed, crenulate to feebly granular; dorsoexternal carina strongly developed, crenuate to feebly granular (male) or weakly developed to faint, smooth (female); externomedian and ventroexternal carinae moderately developed, smooth to slightly crenulate; ventromedian carina weakly to moderately developed, granular (male) or weakly developed, smooth to slightly granular (female); ventrointernal carina weakly to moderately developed, granular. Chela manus rounded (male, female) height greater than width, moderately (male) to sparsely setose (female); dorsal intercarinal surface granular-reticulate (male, female); external intercarinal surface granular-reticulate (male) or reticulate (female) (Fig. 3A, B); dorsal margin strongly developed, granular; digital carina strongly developed, crenulate to smooth (male, female); dorsal secondary carina weakly developed, granular; external secondary carina weakly developed, smooth to slightly granular (male) or weakly developed, smooth (female); ventroexternal carina weakly developed, crenulate; ventromedian carina moderately to strongly developed, coarsely granular to crenulate (male) or crenulate to smooth (female), directed towards midpoint of movable finger articulation; ventrointernal carina weakly developed, smooth to slightly crenulate; internoventral, internomedian and internodorsal carinae weakly developed, slightly granular; internal surface with shallow longitudinal depression where chela rest against patella. Chela fixed finger slightly curved, length equals femur length; dorsal surface smooth and densely setose proximally, external surface flat, internal surface shallowly concave.

*Legs*: Legs I-IV femora and tibiae, prolateral surfaces shagreened; telotarsi, counts of spiniform macrosetae in pro- and retroventral rows (dextral/sinistral): 4/5 5/5:5/5 5/6:6/6 6/6:6/6 6/6 (holotype); basitarsi, spiniform macrosetae pattern: Leg I pst. rst, pm, rm; leg II pt, rt, pst, rst, pm, rm, Rm; legs III-IV pt, rt, vt, rst, vst, vm (holotype).

*Pectines*: Tooth count: 13-14 (male; Fig. 1B) or 11-11 (female)

*Mesosoma*: Tergites I-VI, pre-tergites smooth, post-tergites minutely granular; VII granular. Sternites smooth; dorsosubmedian and dorsolateral carinae weakly to moderately developed, crenulate.

*Metasoma*: Metasomal segments I-V, dorsal intercarinal surfaces shagreened on segments I-IV, smooth on V; lateral intercarinal surfaces shagreened on segments I-IV, smooth on V; ventral intercarinal surfaces smooth on I-V. Segments I-IV, dorsolateral carinae weakly developed, granular; lateral surpamedian carinae moderately developed, granular on I, moderately developed, granular to crenulate on II-III; moderately developed, granular to serrate on IV; lateral inframedian carinae strongly developed, crenulae on I, moderately developed, crenulate to granular on II-IV; ventrolateral carinae strongly developed, crenulate on I, moderately developed, crenulate to slightly granular on II-IV; ventrosubmedian carinae strongly developed, smooth to crenulate on I, moderately developed, smooth to crenulate on II-IV. Segment V length: pedipalp femur length ratio, 1.31 (male), 1.09 (female); dorsolateral carinae strongly developed, crenulate to feebly granular; lateral inframedian carinae moderately to weakly developed, feebly granular on I-III, weakly developed, sparsely granular on IV; ventrolateral carinae moderately developed, granular to feebly serrate, with subspiniform granules; ventromedian carina moderately to strongly developed, granular, with subspiniform granules; ventral transverse carina moderately developed, comprising four subspiniform granules posteriorly; anal arch semicircular; anal subterminal carina moderately developed, comprising twelve subspiniform granules; anal terminal carina vestigial, weakly granular.

*Telson*: Telson, width: length ratio, 0.46 (male), 0.58 (female). Vesicle, lateral surface smooth; ventral surface granular anteriorly. Subaculear tubercle stout, subconical. Aculeus length 1.5.

**Figure 5. F5:**
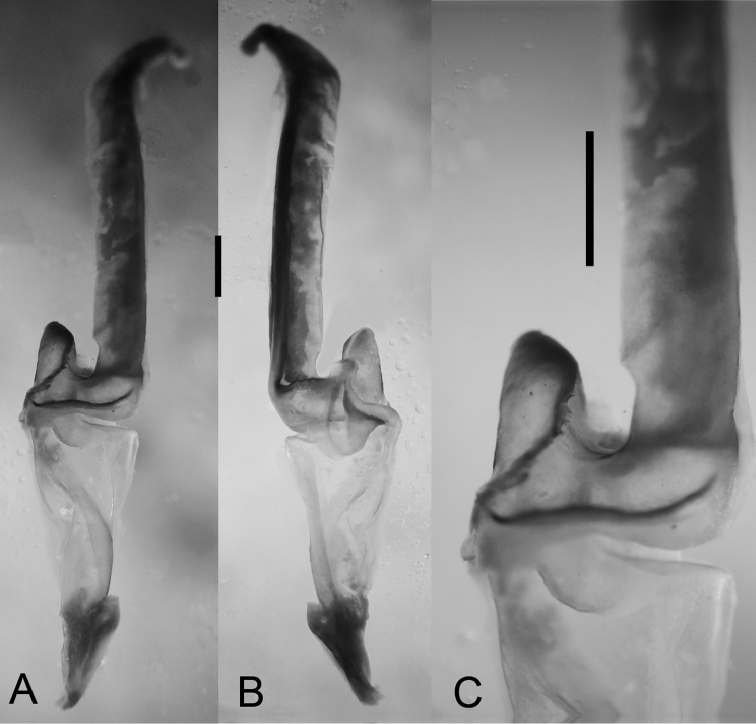
*Diplocentrus franckei* sp. n., paratype ♂ (CNAN) sinistral hemispermatophore. **A** dorsal aspect **B** ental view **C** Capsular region. Scale bars = 1 mm.

*Hemispermatophore*: Lamelliform, weakly sclerotized (Fig. 5); total length 8.9 mm; distal lamella, length 4.8 mm, capsular region width, 1.8 mm; median lobe narrow, margin with small crenulations.

##### Etymology.

This species is dedicated to Dr. Oscar Francke, for his enormous contribution to the taxonomy and systematics of the genus *Diplocentrus*, and his guidance through the years in my education.

##### Distribution.

**Figure 6.**
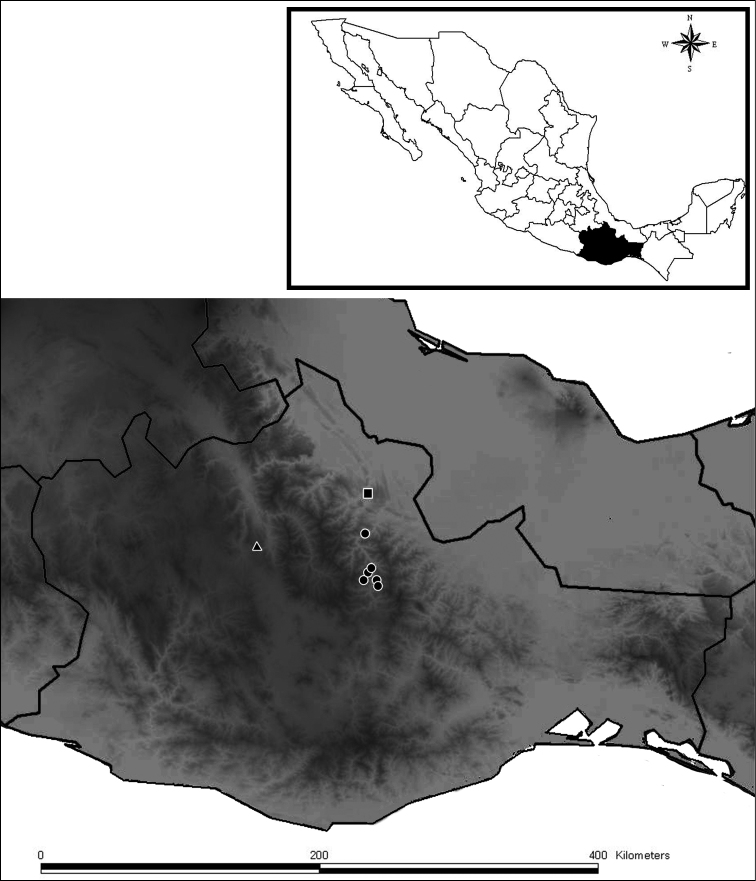
*Diplocentrus franckei* sp. n., known records in Oaxaca (in circles). *Diplocentrus mexicanus* (type locality in triangle). *Diplocentrus jaca* (type locality in square).

*Diplocentrus franckei*, sp. n. is known from the Villa Alta district within the Northern mountain range in Oaxaca; in the municipalities of San Andres Yaa, San Andres Zoolaga, San Juan Tabaa, San Melchor Betaza (Fig. 6).

##### Ecology.

This species was observed first on the soil surface, walking at night with UV detection; later it was collected inside houses, under stones and also in the crevices of rock walls. It was also found doorkeeping at burrow entrances in walls of road cuts. The burrows were constructed at an angle of ca. 30° to the wall, ca. 40–50 cm long and mostly straight with some turns around stones in the soil matrix. The dominant vegetation was the transition between dry tropical forest and pine-oak forest at 1500 m. *Centruroides serrano* Santibáñez-López & Ponce-Saavedra, 2009 was collected in sympatry. The habitat and habitus of *Diplocentrus franckei*, sp. n. are consistent with the pelophilous ecomorphotype (Prendini 2001).

## Supplementary Material

XML Treatment for Diplocentrus franckei
